# Construction of a nomogram risk prediction model for prolonged mechanical ventilation in patients following surgery for acute type A aortic dissection

**DOI:** 10.3389/fcvm.2024.1335552

**Published:** 2024-03-13

**Authors:** Yun Yu, Yan Wang, Fang Deng, Zhigang Wang, Beibei Shen, Ping Zhang, Zheyun Wang, Yunyan Su

**Affiliations:** Department of Cardiac Surgery, Nanjing Drum Tower Hospital, Nanjing, China

**Keywords:** acute type A aortic dissection, prolonged mechanical ventilation time, risk factors, prediction model, nomogram

## Abstract

**Background:**

This study aims to analyze the risk factors associated with prolonged mechanical ventilation (PMV) in patients following surgical treatment for acute type A aortic dissection (ATAAD). The objectives include constructing a predictive model for risk assessment and validating its predictive efficacy.

**Methods:**

A total of 452 patients diagnosed with ATAAD and undergoing surgical procedures at a tertiary hospital in Nanjing between January 2021 and April 2023 were selected using a convenience sampling method. Patients were categorized into two groups: PMV group (*n* = 132) and non-PMV group (*n* = 320) based on the occurrence of prolonged mechanical ventilation (PMV), and their clinical data were compared. The data were randomly divided into a modeling set and a validation set in a 7:3 ratio. Risk factors for PMV were identified in the modeling group using logistic regression analysis. A risk prediction model was constructed using R 4.1.3 software, visualized via a column chart. Receiver Operating Characteristic (ROC) curves were generated using the validation set to assess model differentiation. Calibration curves were plotted to evaluate accuracy and consistency, and Decision Curve Analysis (DCA) was applied to evaluate clinical utility.

**Results:**

The logistic regression analysis identified age, body mass index, preoperative white blood cell count, preoperative creatinine, preoperative cerebral hypoperfusion, and cardiopulmonary bypass time as significant risk factors for postoperative PMV in patients with ATAAD. The area under the curve (AUC) for the validation set ROC curve was 0.856, 95% confidence interval (0.805–0.907), indicating good discrimination. Calibration curves revealed strong alignment with the ideal curve, and the Hosmer-Lemeshow goodness-of-fit test indicated a well-fitted model (*P* = 0.892). The DCA curve demonstrated a high net benefit value, highlighting the model's strong clinical utility.

**Conclusions:**

The risk prediction model developed in this study for PMV in patients undergoing surgery for ATAAD exhibits robust predictive performance. It provides valuable insights for healthcare practitioners in predicting the likelihood of PMV and devising timely and personalized intervention strategies.

## Introduction

Acute type A aortic dissection (ATAAD) stands as one of the most intricate and catastrophic cardiovascular conditions, characterized by its sudden onset and rapid progression (1). Without timely intervention, the mortality rate escalates at a staggering 1%−2% per hour within the initial 48 h of onset (2), with the potential for a 30-day mortality rate as high as 90% (3). Surgical intervention represents the primary treatment modality (4). While advancements in cardiac surgery technology have substantially reduced the operative mortality associated with ATAAD (2, 5), postoperative in-hospital mortality remains relatively high, ranging between 4.67% and 26% (6, 7). Postoperative complications notably contribute to this in-hospital mortality (8).

In contrast to other cardiac surgeries, individuals following ATAAD surgery are more prone to a spectrum of complications, including circulatory instability, bleeding, acute myocardial injury, acute kidney injury, neurological impairment, and respiratory failure (9). The incidence of respiratory failure, notably, is alarmingly high at 51.6% (10). Such complications frequently necessitate prolonged mechanical ventilation (PMV) (>48 h) (11). PMV not only amplifies postoperative complications, prolongs intensive care unit (ICU) and hospital stays but also significantly heightens postoperative mortality rates among ATAAD patients, diminishing their survival prospects post-discharge (12, 13). Accurately identifying patients at high risk for postoperative PMV is thus of paramount importance, enabling the early implementation of personalized care strategies for ATAAD patients.

While effective risk prediction models aid in identifying disease risk, the current research predominantly focuses on preoperative, intraoperative, and postoperative risk factor analysis for PMV after ATAAD surgery (11, 13). As of now, there is no established risk prediction model specifically designed for PMV following ATAAD surgery. Therefore, drawing from both domestic and international literature alongside clinical data, this study endeavors to construct a risk prediction model for PMV subsequent to ATAAD surgery. The primary aim is to provide medical practitioners with a reference tool for early identification of high-risk patients and the timely formulation of preventive strategies.

## Methods and materials

### Study population

The sample size was determined using the Logistic independent variable event number method, ensuring that each predictor included in the final model contained at least 10 positive cases (14). Anticipating the inclusion of 6 factors in this study, a minimum of 60 patients with PMV was deemed necessary. Considering the incidence of PMV post-ATAAD surgery at 28.9% (9), a minimum sample size of 208 cases was calculated. Employing a convenient sampling method, a total of 452 patients who underwent ATAAD surgery in Nanjing Drum Tower Hospital between January 2021 and April 2023 were selected as the research subjects. The inclusion criteria stipulated: (1) Patients aged 18 years or older; (2) Patients who underwent ATAAD surgery. Exclusion criteria comprised: (1) Patients who deceased during the operation or within 24 h post-operation or were discharged automatically; (2) Patients with incomplete data; (3) Patients with preoperative tracheal intubation. This study received approval from the hospital's ethics committee (Approved number: 2022-157-01).

### Surgical procedures

All surgeries were conducted under general anesthesia with the aid of cardiopulmonary bypass (CPB) and performed through a median sternotomy. The establishment of CPB involved the routine cannulation of the femoral artery or right axillary artery in conjunction with the right atrium. Precisely administered cold-blooded cardioplegia fluid was directly injected through the coronary artery opening to ensure myocardial protection. Furthermore, myocardial protection fluid was retrogradely administered through the coronary sinus. The selection of specific surgical procedures was tailored according to the individual extent of aortic dissection and the unique characteristics of aortic root lesions. Subsequent to the surgical interventions, all patients were transferred back to the Intensive Care Unit (ICU) for standardized monitoring and necessary medical attention.

### Respiratory weaning protocol

After the operation, all patients underwent mechanical ventilation using an appropriate pressure control mode to sustain arterial oxygen saturation above 95%. Criteria guiding the weaning process from mechanical ventilation, as outlined in references (9, 11), included achieving full consciousness, maintaining stable hemodynamics without excessive bleeding, ensuring a normal acid-base balance, complete recovery from muscle relaxation, a tidal volume greater than 6 ml/kg, a respiratory rate below 30/min, an oxygenation index exceeding 200 mmHg, and an arterial carbon dioxide partial pressure below 50 mmHg. In cases where patients encountered hypoxemia following extubation, the application of noninvasive positive-pressure ventilation or high-flow oxygen therapy was promptly administered.

### Data collection and quality control methods

This retrospective study encompassed a comprehensive review of previous literature, culminating in the inclusion of a total of 27 risk factors associated with postoperative PMV, categorized into three groups. Class A comprised preoperative data, such as age, gender, body mass index (BMI), hypertension, diabetes, pulmonary disease (including pulmonary infection, bronchiectasis, COPD, asthma and lung cancer), history of cardiovascular surgery, cardiovascular diseases, cerebrovascular diseases, intestinal, limb, and cerebral malperfusion, neutrophil count, platelet count, troponin T, creatinine, urea nitrogen, alanine aminotransferase, serum lactate level, C-reactive protein, interleukin-6 and D-dimer. Class B encapsulated intraoperative data, incorporating CPB time, aortic cross-clamp time, and duration of deep hypothermic circulatory arrest (DHCA), concomitant valve surgery, concomitant coronary artery bypass grafting (CABG), type of aortic arch treatment. Class C included postoperative data, specifically drainage volume 24 h after surgery, ICU stay duration, hospital stay duration, and incidences of reintubation.

Data acquisition was facilitated through the hospital's electronic medical record system and nursing records. The process of data collection, entry, and validation was executed by three cardiac surgery nurses who received uniform training. The collected data underwent a meticulous scrutiny process led by an attending physician and an associate chief nurse to ensure both authenticity and accuracy.

### Statistical analysis

The statistical analysis was conducted using SPSS 22.0 and R 4.1.3. Count data were presented as either case numbers or percentages, with inter-group comparisons performed using the chi-square test (or Fisher's exact test). Measurement data following a normal distribution were described using mean ± standard deviation, and inter-group comparisons were conducted through the two independent sample *t*-test. Skewed distribution data were represented using median and interquartile range, with inter-group comparisons analyzed via the Mann-Whitney *U* test. To eliminate potential baseline confounders, a 1:1 propensity score matching (PSM) was carried out between the two groups. All preoperative variables were included in the analysis. The standardized differences for variables were balanced after the match, with a standardized difference <20% considered an acceptable level of imbalance.

A comparison of clinical data between patients with and without PMV was performed. The dataset was randomly partitioned into a modeling set and a validation set at a 7:3 ratio. Logistic regression analysis was conducted based on the modeling set's data. The R 4.1.3 software was utilized to generate a nomogram and develop the predictive model. The discriminative ability of the model was assessed using the receiver operating characteristic curve (ROC), whereas the calibration curve was used to evaluate the model's accuracy and consistency. The clinical utility of the model was evaluated through decision curve analysis (DCA). Statistical significance was defined at a *P*-value of less than 0.05.

## Results

### General information of the subjects

This study comprised a total of 452 enrolled patients, of which 343 were males (75.88%) and 109 were females (24.12%). The average age of the participants was (53.6 ± 13.8) years, with an average BMI of (25.6 ± 4.3) kg/m^2^. The mean duration of ICU stay was (6.2 ± 5.7) days, while the average hospital stay duration was (17.7 ± 9.8) days. Out of the total participants, 420 cases were successfully discharged, while 32 cases (7.1%) resulted in mortality. PMV was observed in 132 patients, indicating an incidence rate of 29.2%.

### Comparison of clinical data

The comparison between the PMV and non-PMV groups after ATAAD surgery revealed notable differences. Significantly distinctive variations were observed in multiple factors, including age, BMI, history of hypertension, preoperative limb malperfusion, preoperative cerebral malperfusion, preoperative neutrophil count, platelet count, creatinine, D-dimer, CPB time, aortic cross-clamp time, postoperative 24-h drainage volume, ICU stay duration, hospital stay duration, incidences of reintubation, and in-hospital mortality. All these differences demonstrated statistical significance (*P *< 0.05), as detailed in Table 1. After PSM, 129 pairs of patients with comparable baseline characteristics were identified. The in-hospital mortality rates were 14.3% (18 patients) in the PMV group and 3.9% (5 patients) in the control group (*P *= 0.008).

**Table 1:** Comparison of perioperative clinical data of patients with acute type A aortic dissection.

Variables	PMV (*n* = 132)	Non-PMV (*n* = 320)	*P* value
Demographic data			
Age (year)	56.0 ± 14.4	52.5 ± 13.4	0.013
Male (%)	96 (72.7)	247 (77.2)	0.313
BMI (kg/m^2^)	26.5 ± 4.8	25.3 ± 4.1	0.016
Medical history			
Hypertension (%)	111 (84.1)	240 (75.0)	0.035
Diabetes (%)	7 (5.3)	13 (4.1)	0.560
Previous pulmonary diseases (%)	22 (16.7)	44 (13.8)	0.425
Previous cardiovascular surgery (%)	11 (8.3)	17 (5.3)	0.226
Previous cardiovascular disease (%)	22 (16.7)	62 (19.4)	0.501
Previous cerebrovascular disease (%)	17 (12.9)	34 (10.6)	0.491
Intestinal malperfusion (%)	3 (2.3)	7 (2.2)	1.000
Limb malperfusion (%)	9 (6.8)	8 (2.5)	0.034
Cerebral malperfusion (%)	15 (11.4)	7 (2.2)	< 0.001
Preoperative laboratory data			
Neutrophil count (10^9^/L)	11.5 ± 10.2	9.6 ± 4.3	0.007
Hb (g/L)	130.3 ± 20.6	128.8 ± 20.8	0.479
Plt (10^9^/L)	154.5 ± 60.0	177.0 ± 63.3	0.001
TNT (ng/ml)	0.3 ± 1.4	0.3 ± 2.5	0.786
Cr (μmol/L)	103.7 ± 59.1	87.9 ± 68.8	0.023
BUN (mmol/L)	8.0 ± 3.8	7.8 ± 18.9	0.921
ALT (u/L)	65.2 ± 224.4	44.7 ± 117.7	0.228
D-dimer (ng/ml)	30.1 ± 71.2	15.4 ± 47.1	0.014
IL-6 (pg/ml)	192.5 ± 56.6	180.4 ± 73.2	0.115
Lactate (mol/L)	3.3 ± 2.1	3.0 ± 2.1	0.458
CRP (mg/L)	64.3 ± 78.1	62.6 ± 78.7	0.900
Operative data			
CPB time (min)	211.2 ± 61.2	187.1 ± 53.1	< 0.001
Aortic cross-clamp time (min)	152.4 ± 42.4	137.5 ± 42.2	0.001
DHCA time (min)	26.8 ± 11.5	24.6 ± 11.0	0.057
Concomitant valve surgery (%)	2 (1.5%)	3 (0.9%)	0.250
Concomitant CABG (%)	7 (5.3%)	14 (4.4%)	0.130
Arch treatment			
Untreated (%)	5 (3.8)	11 (3.4)	0.940
Hemi-arch replacement (%)	71 (53.8)	122 (55.6)	
Total arch replacement (%)	56 (42.4)	131 (40.9)	
Postoperatve data			
Drainage volume 24 h after surgery (ml)	636.3 ± 579.5	529.0 ± 351.9	0.017
ICU stay time (day)	10.9 ± 7.7	4.3 ± 3.0	< 0.001
Hospital stay time (day)	22.6 ± 14.3	15.7 ± 6.2	< 0.001
Reintubation of the tube (%)	13 (9.8)	11 (3.4)	0.006
In-hospital mortality (%)	18 (13.6)	14 (4.4)	< 0.001

*P* < 0.05 was considered statistically significant.

### Influencing factors of PMV

Univariate analysis was conducted using the modeling set data, where variables displaying statistical differences were subsequently included in Multivariate analysis. The results of the Multivariate analysis revealed that age, body mass index, preoperative neutrophil count, creatinine levels, preoperative cerebral malperfusion, and CPB time were identified as risk factors for Prolonged Mechanical Ventilation (PMV) following surgery for ATAAD (*P* < 0.05). These findings are detailed in Table 2.

**Table 2:** Results of multivariate analysis of postoperative PMV in patients with acute type A aortic dissection.

Variables	Univariate analysis			Multivariate analysis		
	OR	95% CI	*P* value	OR	95% CI	*P* value
Age	1.02	1.00–1.03	0.014	1.06	1.03–1.11	<0.001
BMI	1.06	1.01–1.11	0.011	1.08	1.02–1.13	0.004
Hypertension	1.76	1.05–3.06	0.036			
Neutrophil count	1.08	1.02–1.15	0.010	1.10	1.04–1.18	0.008
PLT	0.99	0.99–1.00	<0.001			
Cr	1.00	1.00–1.01	0.041	1.00	1.00–1.01	0.039
D-dimer	1.011	1.00–1.01	0.027			
Limb malperfusion	2.85	1.07–7.76	0.035			
Cerebral malperfusion	5.73	2.36–15.34	<0.001	6.36	2.44–19.24	<0.001
CPB time	1.01	1.00–1.01	<0.001	1.01	1.00–1.01	<0.001

*P* < 0.05 was considered statistically significant.

### Development of a risk prediction model

**Figure 1:**
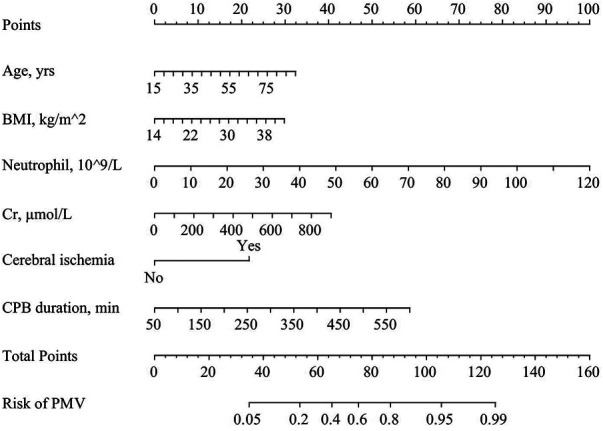
Predictive nomogram for postoperative PMV in patients with acute type A aortic dissection.

A nomogram predicting the risk of PMV following surgery for ATAAD was formulated using the modeling set's data, displayed in Figure 1. In this nomogram, each predictor was allocated a specific score according to the established scoring standard. The total score, which amalgamates the values from the six predictive factors within the model, corresponds to the position on the “PMV risk” axis, indicating the estimated probability of PMV. Higher scores on the nomogram indicate an elevated likelihood of experiencing PMV.

#### Assessment of prediction efficacy and clinical effectiveness analysis of the model

**Figure 2:**
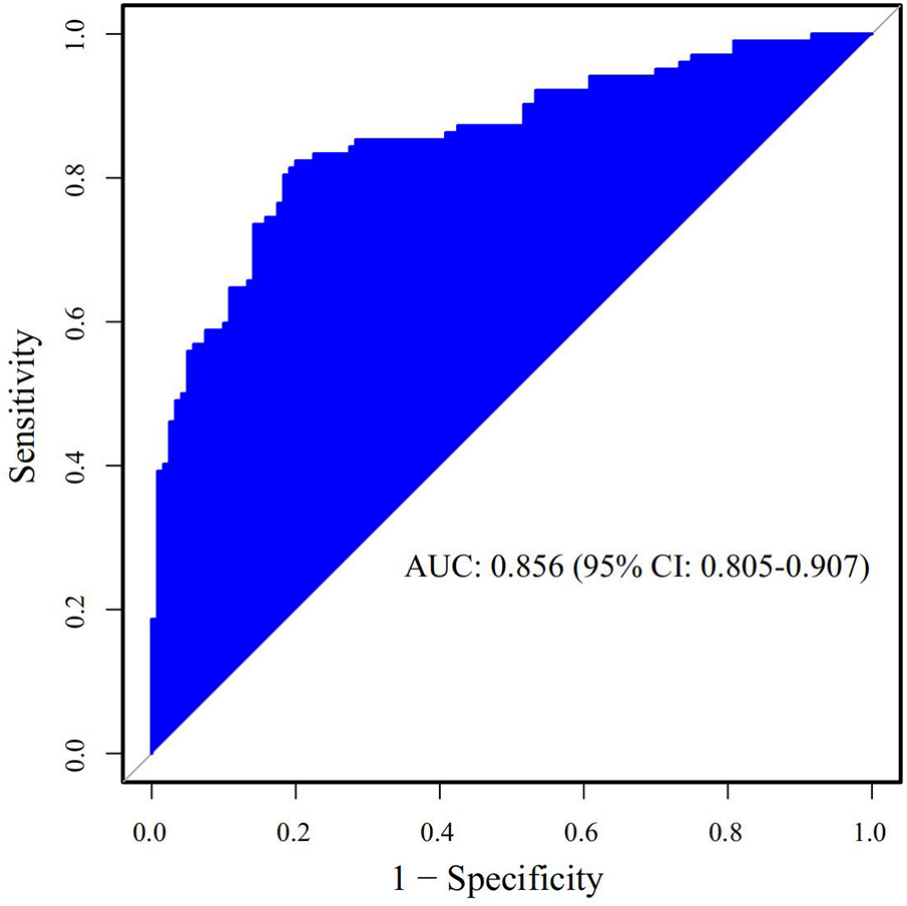
The area under the ROC curve of the prediction model for postoperative prolonged mechanical ventilation in patients with acute type A aortic dissection in the validation set.

**Figure 3:**
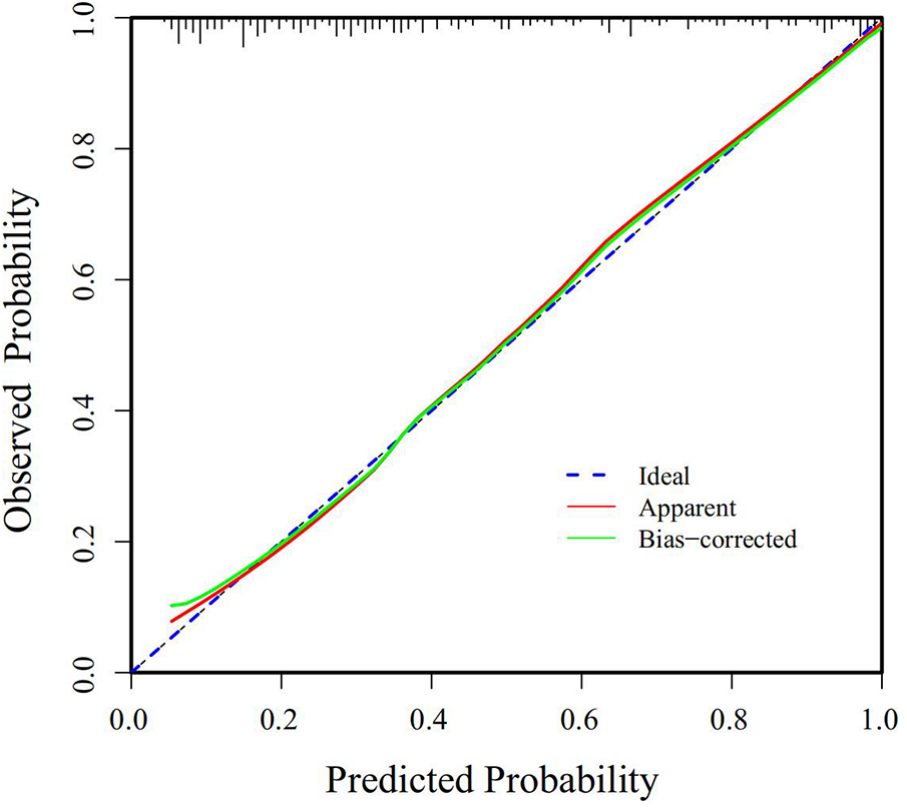
Calibration curve of the predictive model for postoperative PMV in patients with acute type A aortic dissection.

**Figure 4:**
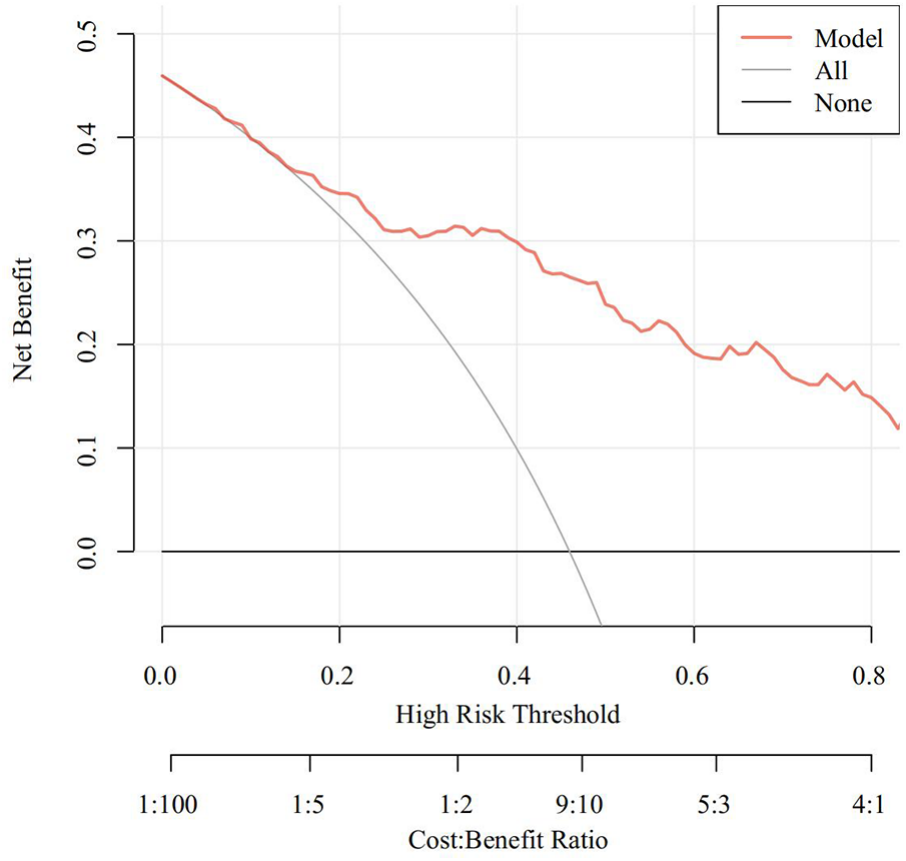
DCA curve of the predictive model for postoperative PMV in patients with acute type A aortic dissection.

The Receiver Operating Characteristic (ROC) curve was generated using the validation set data, with an Area Under the Curve (AUC) of 0.856, 95% CI (0.805–0.907). This AUC value signifies that the model demonstrated commendable discriminatory ability, as depicted in Figure 2. The Calibration Curve illustrated a close alignment with the ideal curve, affirming the model's good fit. The Hosmer-Lemeshow test result of *P *= 0.892 further indicated the model's excellent fit, as visualized in Figure 3. Moreover, the Decision Curve Analysis (DCA) curve, as presented in Figure 4, holistically evaluates the model's performance. DCA focuses on the overall benefit of the model, quantifying the net benefit gained by intervening vs. the potential loss when intervention is not applied in time (15). The curve notably surpasses the reference line within the threshold range of 0–1, suggesting a higher net benefit value, signifying the model's strong clinical utility. This outcome indicates that the nomogram model holds substantial clinical applicability.

## Discussion

PMV is a common complication after ATAAD surgery, and the incidence of PMV varies greatly in different studies. Diaz-Castrillon et al. (12) showed that the incidence of PMV after ATAAD surgery was 18.1%. A retrospective cohort study of 239 patients with ATAAD in Thailand (16) showed that the incidence of PMV was 48.5%. Studies by Chinese scholars (11, 13, 17) showed that the incidence of PMV was 34.7%–44.5%. The incidence of PMV in this study was 29.2%, which was different from that in other studies of China. This may be related to the early implementation of enhanced recovery after surgery in European and American countries, while enhanced recovery after surgery in cardiac surgery in China is still in the exploratory stage (18). The incidence of this study is generally lower than that of most Chinese studies, which may be related to the fact that most of the research data were before 2018, and the exploration of ERAS after cardiac surgery was officially started only when the ERAS Association first released the ERAS guidelines for the perioperative management of cardiac surgery in 2019 (19). In 2019, our center began to establish and implement an enhanced recovery system after surgery (20, 21), improve the extubation strategy, promote early extubation, and reduce the occurrence of PMV to a certain extent. However, the incidence of PMV is still high (29.2%), which affects the prognosis of patients. Therefore, medical staff need to identify the risk factors of PMV after ATAAD surgery, and formulate preventive measures in time to reduce its incidence.

Age is an independent risk factor for PMV following ATAAD surgery, corroborating the findings of Kelly Hu et al. (22) and Trimarchi et al. (23). Elderly patients, compared to younger ones, often present with complicating conditions such as hypertension, diabetes, coronary heart disease, and pulmonary issues, contributing to a more severe condition upon hospital admission, potentially leading to a poorer prognosis (24). Furthermore, the elderly typically possess lower cardiopulmonary reserve, exhibit poorer response to CPB, anesthesia, various medications, and fluid administration, and have a relatively weaker physiological state. These factors can result in heightened heart rate variability, systemic inflammatory status, fluctuations in hormone levels, and diminished immune function (25, 26), collectively increasing the likelihood of various postoperative complications and a higher risk for a poor prognosis. Reddy et al. (27) noted that the risk of PMV increases substantially with age, with a 2.2-fold higher risk in patients aged 65–75 years, a 4.8-fold increase in patients aged 75–80 years, and a 5.5-fold rise in those over 80 years. Consequently, medical practitioners should tailor personalized plans for surgical procedures, drug selection, fluid management, and airway procedures for elderly ATAAD patients to minimize complications and reduce mechanical ventilation duration.

BMI stands as an independent risk factor for PMV and poorer prognoses in patients after ATAAD (24, 28, 29). Lin et al. (24) categorized post-ATAAD patients according to WHO criteria, classifying them into three groups: normal weight (BMI 18.3–23.9 kg/m^2^), overweight (BMI 24–27.9 kg/m^2^), and obese (BMI > 28 kg/m^2^). They noted that the incidence of PMV was higher in the obese group than the overweight group, with the overweight group exhibiting higher rates than the normal weight group. Obese patients accumulate fat in the mediastinum, chest wall, and abdomen, resulting in reduced thoracic volume and limited diaphragm function. These changes decrease lung compliance and functional residual capacity while significantly raising respiratory resistance (30). Simultaneously, these changes can lead to airway hyperresponsiveness by reducing airway smooth muscle tension and shortening the airway smooth muscle (31). Additionally, obese patients have elevated abdominal pressure due to fat accumulation in the abdominal cavity and abdominal wall. During surgical mechanical ventilation, when the diaphragm is entirely relaxed and in a supine anesthesia position, the increased abdominal pressure gets directed to the lung's gravity-dependent area, causing preferential ventilation in certain lung regions, resulting in ventilation and blood flow mismatches and a heightened susceptibility to atelectasis (32). Therefore, healthcare professionals should consider the unique airway characteristics of obese patients in their airway management. This includes adopting lung-protective ventilation strategies (33), reducing airway hyperresponsiveness through appropriate sedation, and encouraging early mobilization to avoid the supine position, thereby enhancing the ratio of ventilation to blood flow.

Preoperative neutrophil count, a marker of non-specific inflammatory response, is a sensitive indicator, and its elevation pre-surgery remains an independent risk factor for PMV, consistent with earlier studies (11, 34). The precise mechanism behind how an increased white blood cell count contributes to PMV remains unclear, but it might be associated with unique features of pulmonary capillaries and the deformability and adhesion molecules of neutrophils (35). Approximately 50%–60% of total circulating neutrophils are stored in the pulmonary circulation's vascular bed, and any disturbance in local neutrophil balance could directly lead to tissue damage (36). Research by Alexiou et al. (37) suggested that using leukocyte filters during CPB could safeguard lung function. Guidelines for enhanced recovery after cardiac surgery (19) propose that judicious use of antibiotics during the perioperative period can avert infection and reduce the duration of mechanical ventilation. Healthcare professionals can anticipate the inflammation level by observing preoperative white blood cell counts, safeguard lung function during surgery, and prudently administer antibiotics during the perioperative period to prevent lung injury or infection. This approach aims to potentially reduce the duration of mechanical ventilation.

Serum creatinine is a widely employed marker for assessing renal function in clinical settings (38). This study exhibited that heightened preoperative serum creatinine levels augmented the risk of PMV in ATAAD patients, aligning with the observations of Eghbalzadeh et al. (39). The potential mechanism revolves around renal function impairment, disrupting normal metabolism and leading to an upsurge in nitrogen-containing substances like creatinine. This, in turn, catalyzes an inflammatory response in the lungs, triggering hypoxemia and subsequently lengthening the duration of mechanical ventilation (40). Wu et al. (41) identified that postoperative renal replacement therapy could mitigate this risk. Medical practitioners can utilize preoperative serum creatinine levels as a predictive measure for patient prognosis and consider early implementation of renal replacement therapy to diminish the likelihood of complications.

The occurrence rate of preoperative cerebral malperfusion among ATAAD patients ranges from 5.2% to 13.1%, displaying clinical symptoms such as coma, transient consciousness loss, stroke, or sensory and language disturbances (42). This phenomenon might arise from brain vessel involvement due to dissection, causing vessel constriction or even blockage, hypoxic encephalopathy from shock, or cerebral embolism due to false lumen thrombosis. This research revealed that preoperative cerebral malperfusion was an independent risk factor for PMV, corroborating the findings of Sultan et al. (43). Lin et al (44) showed that compared with unilateral selective antegrade cerebral perfusion, intraoperative bilateral selective antegrade cerebral perfusion can significantly improve the prognosis of patients with poor cerebral perfusion and shorten the time of mechanical ventilation. Okita et al. (45) suggested that aortic arch branch vessel stent implantation and extracorporeal bypass before central aortic repair could reinstate intravascular perfusion and enhance patient outcomes. Hence, medical practitioners could enhance patient prognosis and curtail the duration of mechanical ventilation by implementing suitable brain protection measures and optimal surgical timing.

This research has revealed that prolonged CPB time stands as an independent risk factor for postoperative PMV in ATAAD patients, aligning with the findings of Maisat et al. (16). Surgical procedures for ATAAD patients are complex and often necessitate lengthy CPB durations. The potential mechanisms contributing to PMV resulting from extended CPB times include pulmonary dysfunction, systemic inflammatory response, cytotoxin generation, embolism, and reperfusion injury (46). Studies have indicated that employing a hemoperfusion device during CPB might reduce the inflammatory response and lung injury (47). Postoperatively, vigilant airway management, lung-protective ventilation strategies, prone positioning, conservative blood transfusions, early extubation, and subsequent noninvasive or high-flow oxygen therapies have proven effective in ameliorating postoperative hypoxia (10, 48), consequently reducing the duration of mechanical ventilation. This study proposes a significant consideration: to minimize CPB duration where possible and promptly implement lung protection measures during and after surgery. Such steps aim to curtail the duration of mechanical ventilation and enhance patient prognoses.

In this study, a risk prediction nomogram model was developed based on patient demographics, objective tests, and test results. Upon model validation, the area under the ROC curve was 0.770, 95% CI [0.720–0.819], signifying a moderate predictive capability suitable for clinical application. The calibration curve demonstrated a close fit to the ideal curve, while the Hosmer-Lemeshow test exhibited a high accuracy with *P* = 0.971. Moreover, the DCA curves surpassed the reference line, indicating a substantial net benefit and a high clinical utility. This model's establishment aids healthcare professionals in promptly evaluating PMV risk following ATAAD surgery, allowing for proactive intervention. This approach reduces the utilization of medical resources and enhances patient prognoses.

## Limitations

Although these predictors reported here have clinical implications, there are some limitations to our work that require further study. First, all patients were from a single tertiary care center, which could lead to the risk of potential selection bias and limit the translation of our results to other centers. Second, detailed intraoperative data on mechanical ventilation, vasopressors and anesthetics were not included in our analysis and we didn't break down specific types and severity of lung disease. Future research can explore other factors which may be correlated with PMV. Besides, our model still needs to be verified in multi-center populations prospectively in the future.

## Conclusions

The risk prediction model developed in this study for PMV in patients after ATAAD surgery exhibits good discrimination and accuracy. It stands to offer valuable guidance for clinical medical practitioners in predicting the risk of PMV and devising tailored preventive strategies promptly. This strategy would allow for the refinement and optimization of the risk prediction model for better generalizability and applicability in clinical practice.

## Data Availability

The original contributions presented in the study are included in the article/Supplementary Material, further inquiries can be directed to the corresponding author.
